# Deacetylation of H4-K16Ac and heterochromatin assembly in senescence

**DOI:** 10.1186/1756-8935-5-15

**Published:** 2012-08-29

**Authors:** Kévin Contrepois, Jean-Yves Thuret, Régis Courbeyrette, François Fenaille, Carl Mann

**Affiliations:** 1 CEA, iBiTecS, Service de Biologie Intégrative et de Génétique Moléculaire (SBIGeM), F-91191, Gif-sur-Yvette, France; 2 CEA, iBiTecS, Service de Pharmacologie et d’Immunoanalyse (SPI), F-91191, Gif-sur-Yvette, France; 3 SBIGeM-Bât. 142, CEA Saclay, F-91191, Gif-sur-Yvette, France

## Abstract

**Background:**

Cellular senescence is a stress response of mammalian cells leading to a durable arrest of cell proliferation that has been implicated in tumor suppression, wound healing, and aging. The proliferative arrest is mediated by transcriptional repression of genes essential for cell division by the retinoblastoma protein family. This repression is accompanied by varying degrees of heterochromatin assembly, but little is known regarding the molecular mechanisms involved.

**Results:**

We found that both deacetylation of H4-K16Ac and expression of HMGA1/2 can contribute to DNA compaction during senescence. SIRT2, an NAD-dependent class III histone deacetylase, contributes to H4-K16Ac deacetylation and DNA compaction in human fibroblast cell lines that assemble striking senescence-associated heterochromatin foci (SAHFs). Decreased H4-K16Ac was observed in both replicative and oncogene-induced senescence of these cells. In contrast, this mechanism was inoperative in a fibroblast cell line that did not assemble extensive heterochromatin during senescence. Treatment of senescent cells with trichostatin A, a class I/II histone deacetylase inhibitor, also induced rapid and reversible decondensation of SAHFs. Inhibition of DNA compaction did not significantly affect the stability of the senescent state.

**Conclusions:**

Variable DNA compaction observed during senescence is explained in part by cell-type specific regulation of H4 deacetylation and HMGA1/2 expression. Deacetylation of H4-K16Ac during senescence may explain reported decreases in this mark during mammalian aging and in cancer cells.

## Background

Genomic DNA in eukaryotes is packaged into chromatin. The histones and non-histone proteins of chromatin compact the DNA and govern its accessibility to enzymes during transcription, replication, repair and recombination. Poorly transcribed regions of the genome are typically found in highly compacted DNA as heterochromatin, whereas transcribed sequences are found in the more accessible euchromatin [1]. Post-translational modifications of histones represent an important mechanism modulating the accessibility of chromatin and contributing to the recruitment of other proteins to chromatin [2]. Histones are extensively modified principally by acetylation, methylation, ubiquitylation, and phosphorylation. Euchromatin is enriched in histones acetylated at lysine residues. Acetylation of H4-K16 plays a particularly important, evolutionarily conserved role in regulating chromatin compaction [3]. Positively charged H4-K16 can form a salt bridge with acidic patches of H2A and H2B on adjacent nucleosomes and thereby contribute to folding of the chromatin fiber in vitro [4-6]. Acetylation of H4-K16 neutralizes its basic charge and inhibits compaction of the chromatin. Acetylation of H4-K16 can also recruit specific bromodomain-containing proteins to chromatin to stimulate transcription [7]. Consistent with these attributes, unacetylated H4-K16 is generally associated with transcriptional silencing and heterochromatin, whereas H4-K16Ac is generally associated with euchromatin [3]. In budding yeast, heterochromatin is formed by the localized action of the Sir2 NAD-dependent histone deacetylase that specifically deacetylates H4-K16Ac to allow binding of the SIR silencing complex [8]. In mammals, X chromosome dosage compensation involves heterochromatization and transcriptional silencing of one of two copies of the X chromosome in female cells. H4-K16 is hypoacetylated on this inactive X chromosome [9]. In contrast, in *Drosophila*, X chromosome dosage compensation involves hyperacetylation of H4-K16 on the single male X chromosome by the MOF (MYST1/KAT8) histone acetyltransferase to increase its transcriptional output relative to the two female X chromosomes [10].

In mammals, several members of the MYST family of histone acetyltransferases are able to acetylate H4-K16, but MOF is principally responsible for the global levels of H4-K16Ac [11,12]. Homozygous MOF knockout mice die during early embryogenesis [13]. Depletion of MOF in mouse or human fibroblasts leads to striking decreases in H4-K16Ac accompanied by abnormal nuclear and mitotic figures [12,14]. Acetylation of H4-K16 by MOF is important for activation of checkpoint pathways in response to DNA damage and for efficient DNA repair [12,14,15]. Global acetylation of H4-K16 in mammals is countered by both class I (HDAC1 and HDAC2) and class III histone deacetylases (SIRT1 and SIRT2) in different contexts [16-18]. HDAC1/2 and SIRT1 are largely nuclear enzymes. In contrast, SIRT2 shuttles continuously between the nucleus and cytoplasm, but is largely cytoplasmic at steady state during the interphase of the cell cycle [19]. SIRT2 has an important role in globally deacetylating H4-K16Ac during mitosis [16].

Heterochromatin assembly is associated with many forms of cellular senescence [20]. Senescence is a stress response of mammalian cells induced by numerous stimuli including telomere loss, oncogene activation, and genotoxic agents [21]. Senescent cells are metabolically active, but do not proliferate in response to mitogenic stimuli. Accumulating evidence shows that senescence has a critical role in tumor suppression, wound healing, and aging *in vivo*[22]. Although a biomarker totally specific to senescent cells has not been identified, senescent human cells often display some common characteristics including cell cycle arrest mediated by the p53 and/or Rb tumor suppressor pathways, morphological changes, induction of SA-β-galactosidase activity, enhanced secretion of some cytokines and metalloproteases, and heterochromatin assembly that may include the formation of highly compacted DNA in the form of senescence-associated heterochromatin foci (SAHFs) [21,22]. SAHFs are facultative heterochromatin that are specifically enriched in the transcriptionally silent H3K9Me3 histone mark, whereas euchromatic marks such as H3K9Ac and H3K4Me are largely excluded from these foci [23]. HMGA1, HMGA2, HP1, and macro-H2A were found to be associated with SAHFs, whereas some histone H1 variants were depleted from the chromatin of senescent cells [24-26]. HMGA1 and the histone chaperone ASF1a have been implicated in the induction of fibroblast senescence and the assembly of heterochromatin, but their precise functions are not known [24,26]. Given the dramatic chromatin reorganization/compaction observed in senescent cells, we postulated that some abundant histone post-translational modifications (PTMs) could vary to favor SAHFs formation. We discovered that a significant fraction of H4-K16Ac was deacetylated in senescence and contributed to heterochromatin assembly and maintenance in fibroblast lines that were competent to form SAHFs.

## Results

### Loss of H4-K16 acetylation is specific to senescent cells and correlates with DNA compaction

**Figure 1:**
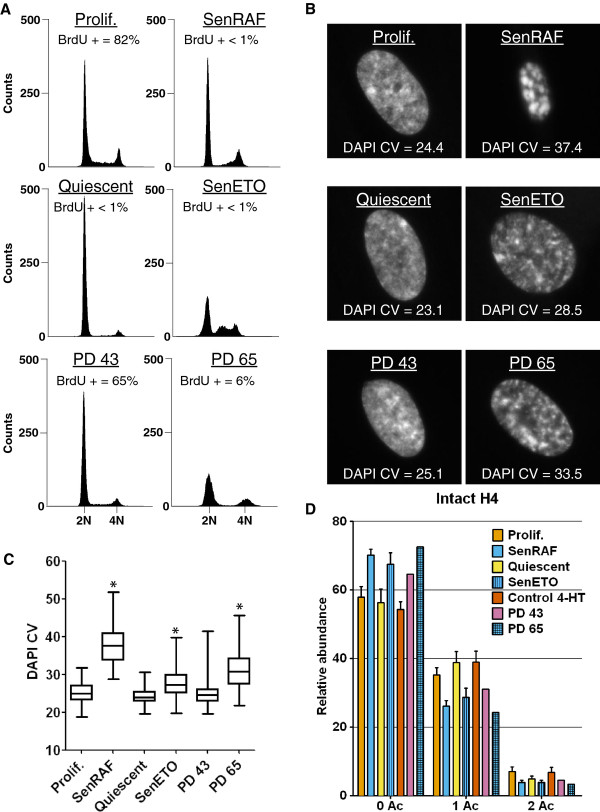
**Loss of H4-K16 acetylation is specific to senescent cells and correlates with DNA compaction. A)** DNA content by flow cytometry. The percentage of cells incorporating bromodeoxy-uridine (BrdU) after 24 hours of incubation is indicated. **B)** DNA 4',6'-diamidino-2-phenylindole (DAPI) staining of representative nuclei and corresponding DAPI coefficient of variation (CV) values. **C)** Boxplots of DAPI CV (n > 70, from one representative experiment of two biological replicates). *DNA compaction statistically different from Prolif. or PD 43 (*P* < 10^-5^, Welch *t*-test). **D)** Relative abundance of H4 acetylation states measured at the protein level on deconvoluted mass spectra (Figure 3). Error bars show SD of at least three biological replicates. Prolif.: proliferating WI-38hTERT/GFP-RAF-ER; SenRAF: WI-38hTERT/GFP-RAF-ER + 20 nM 4-HT (5 days); Quiescent: serum-starved WI-38hTERT/GFP-RAF-ER (5 days); SenETO: WI-38hTERT/GFP-RAF-ER + 20 μM etoposide (5 days); Control 4-HT: WI-38hTERT + 20 nM 4-HT (5 days); PD 43: population doubling 43, proliferating WI-38 cells; PD 65: replicatively senescent WI-38 population.

**Figure 2:**
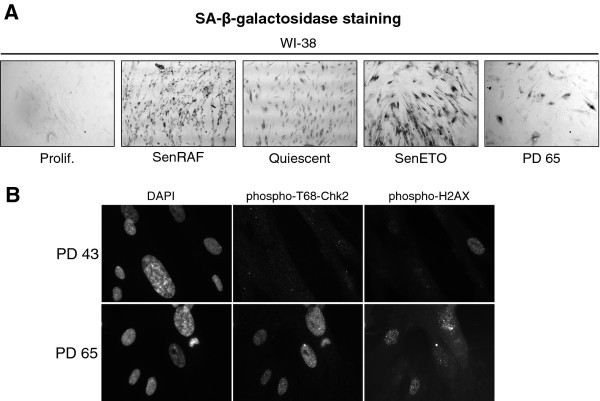
**A) Bright field pictures of WI-38 fibroblasts showing SA-β-galactosidase activity assayed as described**[29]**. B)** Fluorescent microscopy images of WI-38 fibroblasts showing 4',6'-diamidino-2-phenylindole (DAPI), phospho-T68-Chk2 and phospho-H2AX staining in proliferating (PD 43) and replicatively senescent cells (PD 65).

Heterochromatin assembly has been associated with many forms of cellular senescence. In the most extreme cases, senescence induced by oncogene expression is accompanied by the formation of striking SAHFs that are readily visible by 4′,6′-diamidino-2-phenylindole (DAPI) staining. For example, expression of an activated RAS oncogene (RASval12) induces senescence with SAHFs in many human fibroblasts [23]. Moreover, expression of an activated oncogenic form of the B-RAF kinase (B-RAF-V600E) induces senescence with SAHFs in human melanocytes [27]. Such highly condensed masses of heterochromatin are not normally seen in proliferating human cells aside from the Barr body (inactive X chromosome) in female cells. We used mass spectrometry (MS) protein profiling [28] to monitor many histone variants and their modification by acetylation, methylation, or phosphorylation. We compared histone profiles from proliferative, quiescent and senescent cells. Senescence was induced by three different stresses: expression of an activated form of the RAF1 (C-RAF) kinase, telomere shortening (replicative senescence) and genotoxic stress (etoposide, a topoisomerase II inhibitor that provokes DNA double-strand breaks). We monitored bromodeoxy-uridine (BrdU) incorporation, FACS (Fluorescence Activated Cell Sorting) profiles, and SA-β-galactosidase expression in each of these experimental conditions to confirm the expected effects of each treatment on cell proliferation, cell cycle profiles, and senescent marker expression (Figures 1A and 2A).

Our reference population was proliferating hTERT-immortalized WI-38 human embryonic lung fibroblasts grown in 5% oxygen. These cells are free from stress engendered by telomere attrition or growth under hyper-physiological 20% ambient oxygen. They also expressed an activated form of the RAF1 kinase fused to green fluorescent protein (GFP) and the estrogen receptor domain (GFP-RAF-ER) that could be activated with 4-hydroxy-tamoxifen (4-HT). Activation of the RAF1 kinase in these cells leads to a rapid hyper-stimulation of the ERK1/2 mitogen-activated protein kinase pathway that induces senescence accompanied by striking SAHFs within 3 days [29]. We prepared chromatin from WI-38hTERT/GFP-RAF-ER cells treated with 4-HT for 5 days, at which time prominent SAHFs had been well established for 2 days (Figure 1B). This sample allowed us to search for modifications of chromatin in senescent cells with highly compacted heterochromatin. Chromatin compaction in quiescent cells was not distinguishable from that of proliferating cells (Figure 1B,C) using the coefficient of variation (CV) of DAPI staining (DAPI CV) within the nucleus as a quantitative metric of DNA compaction in individual cells (see the Methods section). The chromatin of senescent cells treated with etoposide was less highly compacted than for RAF-induced senescence (Figure 1B,C). Chromatin from proliferating non-immortalized WI-38 fibroblasts at population doubling (PD) 43, and from a culture at PD 65, was also prepared. Most PD 65 cells had experienced replicative senescence as shown by the inhibition of BrdU incorporation (Figure 1A) and SA-ß-gal staining (Figure 2A). PD 65, but not PD 43 cells, also stained strongly positive for phospho-H2AX and phospho-T68-Chk2 (Figure 2B), markers of an activated DNA damage checkpoint, as expected for replicatively senescent cells containing defective telomeres [30]. Chromatin in senescent PD 65 cells was more compact than in cells treated with etoposide for 5 days, but less than in cells induced into senescence by active RAF1 for 5 days (Figure 1B,C).

**Figure 3:**
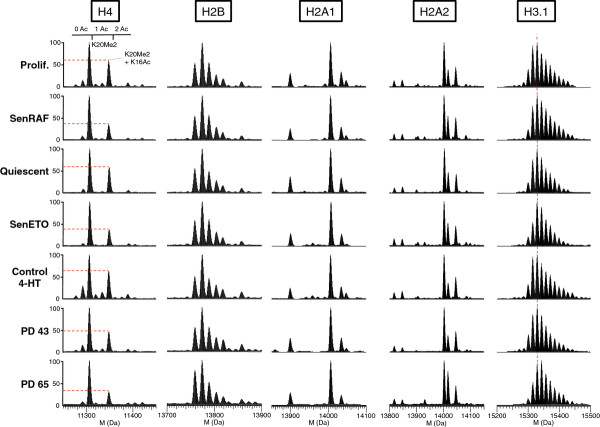
**Deconvoluted mass spectra of intact core histones H4, H2B, H2A1, H2A2 and H3.1 extracted from WI-38 fibroblasts under the indicated experimental conditions.** For each acetylation state of H4, unmodified, mono- and di-methylated K20 forms are visible.

Analysis of the histone variants and PTMs by our profiling method revealed surprisingly few differences in the relative abundance of the predominant histone isoforms when comparing the different samples (Figure 3). However, we noticed that H3.1 was more highly modified by methylation and/or acetylation in non-proliferating cells (senescence and quiescence) compared to cycling cells. Since this effect was not specific to senescent cells, we did not pursue the analysis of H3.1.

**Figure 4:**
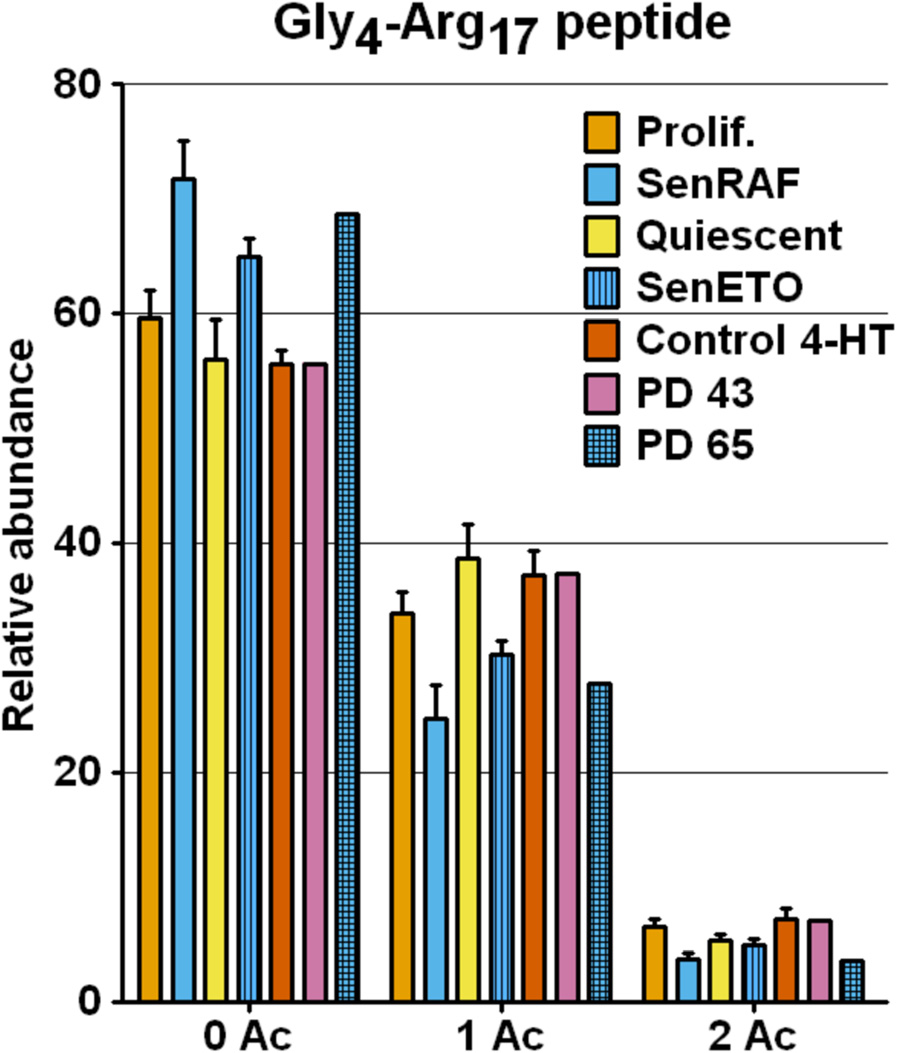
**Relative abundance of H4 acetylation states measured on the Gly**_**4**_**-Arg**_**17**_**peptide by mass spectrometry (MS) analyses.** Error bars show SD of at least three biological replicates. Prolif.: proliferating WI-38hTERT/GFP-RAF-ER; SenRAF: WI-38hTERT/GFP-RAF-ER + 20 nM 4-HT (5 days); Quiescent: serum-starved WI-38hTERT/GFP-RAF-ER (5 days); SenETO: WI-38hTERT/GFP-RAF-ER + 20 μM etoposide (5 days); Control 4-HT: WI-38hTERT + 20 nM 4-HT (5 days); PD 43: population doubling 43, proliferating WI-38 cells; PD 65: replicatively senescent WI-38 population.

The most striking effect we observed was a significant and specific decrease in histone H4 lysine acetylation in senescent cells (Figures 1D and 3). In proliferating cells, approximately 55 to 60% of H4 was unacetylated on lysine, approximately 35 to 40% was mono-acetylated, and approximately 5 to 10% was di-acetylated. In senescent cells, H4-lysine mono-acetylation was decreased by at least 25 to 30% (Figure 1D). Although this represents a modest relative decrease, in absolute amounts it represents the very substantial deacetylation of 6 million molecules of H4 per cell (A diploid human cell contains approximately 30 million nucleosomes and thus 60 million molecules of H4, of which about 24 million molecules are acetylated in proliferating cells and 18 million molecules in senescent cells). This decreased acetylation was confirmed by MS analyses on the Gly_4_-Arg_17_ tryptic peptide (Figure 4). H4 acetylation varies during the cell cycle, being lower in M/G1 and increasing in S/G2 cells [16,31,32]. H4 deacetylation in senescence could thus be an indirect consequence of the proliferative arrest. However, decreased H4 lysine acetylation was not detected in quiescent cells (Figure 1D) that were similarly blocked in their proliferation (Figure 1A), indicating that decreased H4 acetylation is specific to the senescent state.

### Deacetylation of H4 mainly occurs on K16 during senescence

**Figure 5:**
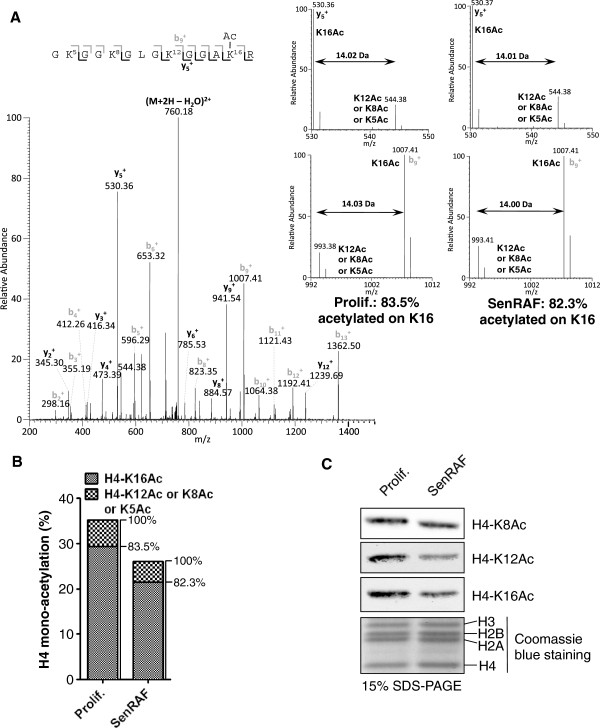
**H4 is mainly deacetylated on K16. A)** MS/MS spectrum of the mono-acetylated H4 tryptic peptide Gly_4_-Arg_17_ (m/z 768.946) from a SenRAF sample. Two rounds of propionylation were performed before and after trypsin digestion to propionylate free unmodified lysines and N-termini. Consequently, the 14 Da differences shown in y_5_^+^ and b_9_^+^ fragment ions represent the mass difference between acetylated and propionylated lysine. The spectrum is a mean of ten experiments along the chromatographic peak. Acetylation on K16 is calculated based on the ratio of the specific daughter ions y_5_^+^ and b_9_^+^ shown as insert in Prolif. and SenRAF samples. K16Ac abundance is the mean of three independent biological replicates (mean coefficient of variation, CV = 3.2%). **B)** Relative distribution of lysine acetylation on mono-acetylated H4 calculated from the MS/MS spectra on Gly_4_-Arg_17_ tryptic peptides. The relative abundances of H4 mono-acetylation come from Figure 1D. **C)** Immunoblot showing the levels of H4-K8Ac, H4-K12Ac and H4-K16Ac in acid-extracted histones. Loading control: Coomassie blue staining of histones. Prolif.: proliferating WI-38hTERT/GFP-RAF-ER; SenRAF: WI-38hTERT/GFP-RAF-ER + 20 nM 4-HT.

H4 can be acetylated on four lysine residues: K5, K8, K12 and K16 [33]. We used MS/MS analysis of the H4 Gly_4_-Arg_17_ tryptic peptide to identify the lysine residues that are preferentially deacetylated during RAF senescence. Previous work showed that the relative distribution of acetylated lysine residues could be determined by measuring the ratio of specific MS/MS peptide fragments [34]. In the fraction of mono-acetylated H4 Gly_4_-Arg_17_ peptides, 83.5% and 82.3% were acetylated on K16 in proliferating and senescent cells, respectively (Figure 5A,B). Most of the remaining peptides were acetylated on K12. Since the relative ratio of positional acetyl-lysine isoforms of H4 is similar in proliferating and senescent cells, the deacetylation observed in senescence must occur in relative proportion to the abundance of these isoforms. This conclusion was independently confirmed by immunoblotting using antibodies specific for H4-K16Ac and H4-K12Ac (Figure 5C). In contrast, the acetylation of H4-K8 did not vary significantly between proliferating and senescent cells.

### MOF and SIRT2 balance contributes to the H4-K16 acetylation level and DNA compaction during RAF-induced senescence

**Figure 6:**
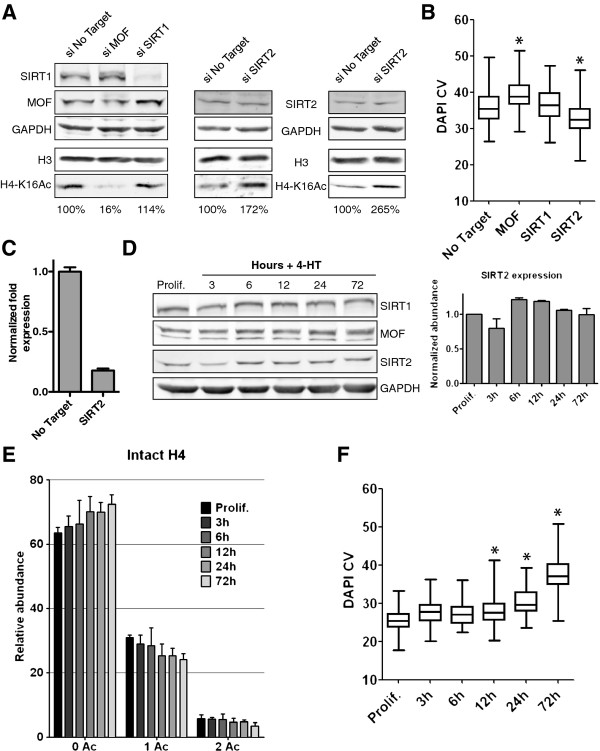
**SIRT2 contributes to deacetylation of H4-K16Ac and DNA compaction during RAF-induced senescence. A)** Immunoblot of extracts from WI-38hTERT/GFP-RAF-ER cells treated with siRNA (24 hours) and 20 nM 4-HT (3 days) using anti-SIRT1, -SIRT2, -MOF, -GAPDH (loading control), -H4-K16Ac, -H3 (loading control) antibodies. Two independent experiments are shown for the depletion of SIRT2. **B)** Boxplots of 4',6'-diamidino-2-phenylindole coefficient of variation (DAPI CV) (n > 70, from one experiment). *DNA compaction statistically different from No Target (*P* < 10^-5^, Welch *t*-test). Biological replicates are shown Figure 7B. **C)** mRNA level of SIRT2 by qPCR after siRNA treatment (24 hours) and 20 nM 4-HT (6 hours). **D)** Immunoblot of WI-38hTERT/GFP-RAF-ER cells treated with 20 nM 4-HT for the indicated times using anti-SIRT1, -SIRT2, -MOF and -GAPDH (loading control) antibodies. Histogram shows the normalized level of SIRT2 to GAPDH for two independent time courses. **E)** Relative abundance of H4 acetylation states measured at the protein level on deconvoluted mass spectra. Error bars show SD of three biological replicates. **F)** Boxplots of DAPI CV (n > 60, from one experiment). *DNA compaction statistically different from Prolif. (*P* < 10^-5^, Welch *t-*test). Prolif.: proliferating WI-38hTERT/GFP-RAF-ER.

**Figure 7:**
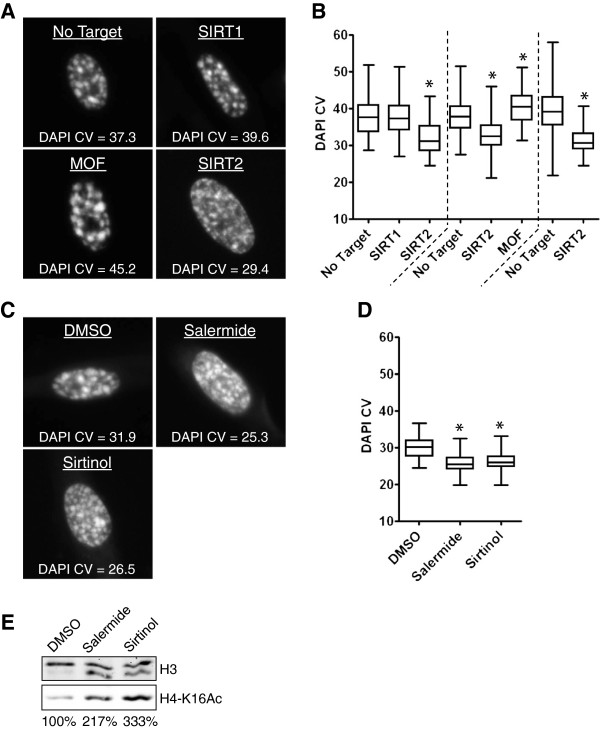
**Effect of SIRT1, SIRT2, and MOF depletion, and sirtuin inhibitors on DNA compaction during RAF-induced senescence of WI-38hTERT/GFP-RAF-ER. A)** DNA 4',6'-diamidino-2-phenylindole (DAPI) staining of representative nuclei and corresponding DAPI coefficient of variation (CV) values (from Figure 6B). **B)** Boxplots of DAPI CV (n > 60, three experiments are shown). *DNA compaction statistically different from No Target (*P* < 10–5, Welch *t*-test). **C)** DNA DAPI staining of representative nuclei and corresponding DAPI CV values. **D)** Boxplots of DAPI CV (n > 80, from one experiment). *DNA compaction statistically different from solvent DMSO control (*P* < 10–5, Welch *t*-test). **E)** Immunoblot showing the level of H4-K16Ac in acid-extracted histones. Loading control: H3. DMSO: WI-38hTERT/GFP-RAF-ER + 0.1% dimethyl sulfoxide (DMSO) + 20 nM 4-HT (3 days); Salermide: WI-38hTERT/GFP-RAF-ER + 50 μM salermide + 20 nM 4-HT (3 days); Sirtinol: 25 μM sirtinol + 20 nM 4-HT (3 days).

Remarkably, deacetylation of H4-K16Ac is implicated in transcriptional silencing and heterochromatin formation from yeast to man [5,35]. We thus explored its role in heterochromatin assembly during RAF-induced senescence of human fibroblasts. Histone acetylation has a rapid turnover due to the highly dynamic equilibrium between histone acetyl transferase (HAT) and histone deacetylase (HDAC) activities [36]. Previous work suggested that MOF (MYST1/KAT8) is the key HAT responsible for the bulk global acetylation of H4-K16 in mammals [11,12]. The importance of MOF activity in global H4-K16 acetylation and DNA compaction was confirmed by mRNA depletion concomitant with the induction of RAF-induced senescence. Under these conditions, we observed an enhanced loss of H4-K16 acetylation during senescence (Figure 6A) accompanied by increased DNA compaction and even more prominent SAHFs (Figures 6B and 7A,B). We saw no decrease in MOF levels during RAF-induced senescence of WI-38hTERT fibroblasts (Figure 6D). However, we cannot rule out that the activity of the MOF complex is inhibited by some other mechanism during cellular senescence.

We next sought to determine the role of HDACs in H4-K16Ac deacetylation during senescence. Previous work indicates a complex situation in which several distinct HDACs including SIRT1 [17], SIRT2 [16], HDAC1 and HDAC2 [18] may contribute to deacetylation of H4-K16Ac depending on the experimental context. Highly effective depletion of SIRT1 levels by siRNA treatment did not inhibit deacetylation of H4-K16Ac or DNA compaction in RAF-senescent cells (Figures 6A,B and 7A,B). Moreover, we saw no significant and reproducible change in SIRT1 levels during RAF-induced senescence (Figure 6D). Thus, SIRT1 is not an important global regulator of H4-K16Ac and DNA compaction under these conditions. In contrast, we noticed a slight but reproducible increase in SIRT2 levels at early times (6–24 h) after GFP-RAF-ER kinase activation by 4-HT (Figure 6D). Interestingly, this early slight increase in SIRT2 level was correlated with a similarly precocious and gradual decrease in H4 acetylation and increase in DNA compaction (Figure 6E,F).

We tested the effect of SIRT2 depletion by siRNAs on the level of H4-K16Ac deacetylation and DNA compaction during RAF-induced senescence. SIRT2 mRNAs were successfully depleted by approximately 80% (Figure 6C). Despite the clear decrease in SIRT2 mRNA, depletion at the protein level was modest (Figure 6A). In our model of RAF-induced senescence, cell division is inhibited within one cell doubling [29], thus limiting the possibility of dilution of the protein over time. The poor depletion of SIRT2 protein suggests that it is quite stable in these conditions. Despite the weak depletion, an increase of H4-K16Ac was reproducibly observed (Figure 6A) and was correlated with a significantly lower DNA compaction in the SIRT2 siRNA-treated cells (Figures 6B and 7A,B). SIRT2 depletion did not affect the induction of senescence by activated RAF kinase (Table 1). It is striking that modest decreases in MOF or SIRT2 led to easily visible effects on H4-K16 acetylation and DNA compaction, whereas strong depletion of SIRT1 had no discernable effect on global H4-K16 acetylation or DNA compaction.

**Table 1:** Depletion of SIRT2 mRNAs or treatment with sirtuin inhibitors does not induce senescence of proliferating cells and does not inhibit RAF-induced senescence as determined by 5-bromo-2'-deoxyuridine (BrdU) incorporation

**Experiment**	**Insult**	**4-HT (20 nM)**	**BrdU-positive cells (%)**
siRNA	No Target	+	< 1
		-	75
	SIRT2	+	< 1
		-	89
Sirtuin inhibitors	DMSO	+	< 1
	(1/1000)	-	89
	Salermide	+	< 1
	(50 μM)	-	18
	Sirtinol	+	< 1
	(25 μM)	-	29

WI-38hTERT/GFP-RAF-ER cells were treated with the indicated siRNAs or sirtuin inhibitors in the presence or absence of 20 nM 4-HT for 3 days and then with fresh medium for 4 days. Cells treated with siRNAs were incubated with BrdU for 24 hours and cells treated with sirtuin inhibitors for 3 days to determine the percentage of cells able to enter S phase under these conditions.

We confirmed a role for sirtuins in H4-K16Ac deacetylation and DNA compaction by treating cells with the sirtuin inhibitors sirtinol and salermide during RAF-induced senescence. Sirtinol and salermide inhibit both SIRT1 [37] and SIRT2 [38]. Neither of these molecules inhibited the RAF-induced proliferative arrest (Table 1), but they both blocked deacetylation of H4-K16Ac and inhibited DNA compaction in the RAF-senescent cells (Figure 7C,D,E). Since SIRT1 depletion had no effect on these processes, it seems likely that chemical inhibition of SIRT2 produced an effect that was similar to mRNA depletion of SIRT2.

**Figure 8:**
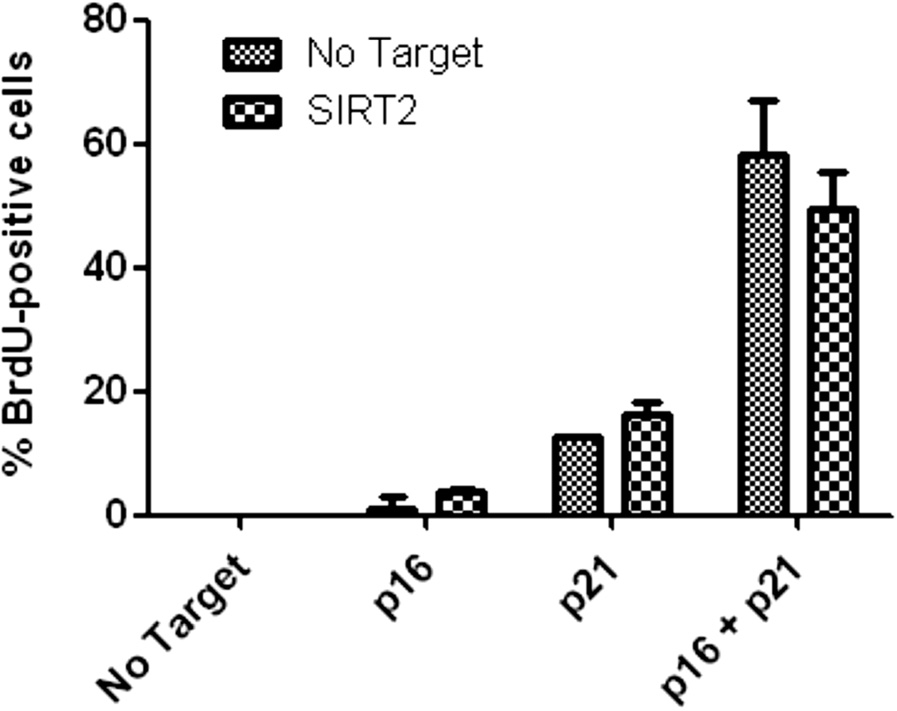
**Effect of SIRT2 depletion on the reversion of RAF-induced senescence after cyclin-dependent kinase inhibitor (CKI) knockdown.** WI-38hTERT/GFP-RAF-ER cells were transfected (24 hours) and induced into senescence by treatment with 20 nM 4-HT for 3 days. Senescent cells were then transfected with the indicated siRNAs and cells were incubated for a further 3 days before testing their capacity to incorporate 5-bromo-2'-deoxyuridine (BrdU) during an additional 24-hour incubation. Error bars show SD of two biological replicates.

We also tested the effect of SIRT2 siRNA treatment on the stability of the senescent state. WI-38hTERT/GFP-RAF-ER cells were transfected with SIRT2 or No Target siRNAs and then induced into senescence by treatment with 20 nM 4-HT for 3 days. Senescent cells were then transfected with No Target siRNA or siRNAs to p16, or p21, or p16 + p21, and cells were incubated for a further 3 days before testing the maintenance of senescence by the capacity of cells to incorporate BrdU during an additional 24-hour incubation. We previously showed that knockdown of p16, or p21, or p16 + p21 can partially reverse RAF-induced senescence [29]. Despite decreased DNA compaction, we observed no significant increase in reversion of RAF-senescent cells treated with SIRT2 siRNAs (Figure 8). Thus, SIRT2 contributes to assembling senescence-associated heterochromatin, but may not be required for maintenance of the senescent state.

### Class I HDACs contribute to deacetylation of H4-K16Ac and DNA compaction in senescent cells

**Figure 9:**
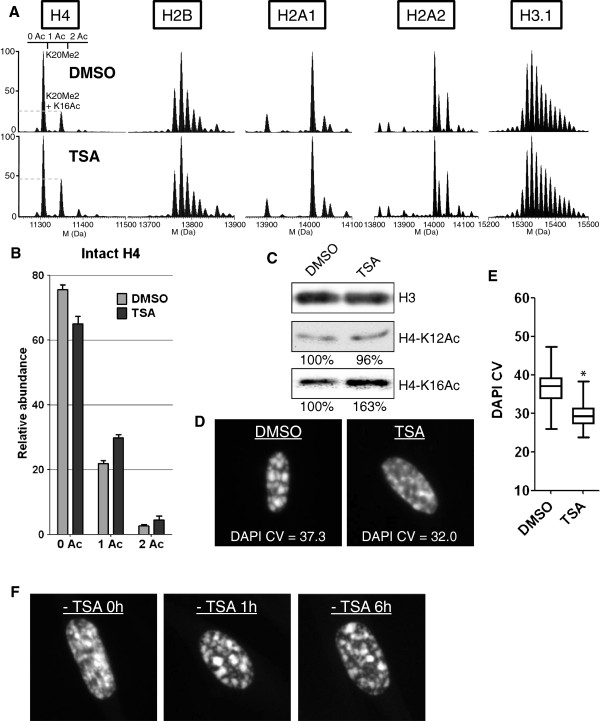
**Treatment of senescent cells with trichostatin A** (**TSA) increases H4-K16Ac and decreases DNA compaction in a rapid and reversible manner. A)** Deconvoluted mass spectra of intact core histones in senescent cells treated with dimethyl sulfoxide (DMSO) or TSA for 24 hours. For each acetylation state of H4, unmodified, mono- and di-methylated K20 forms are visible. **B)** Relative abundance of H4 acetylation states measured at the protein level on deconvoluted mass spectra (Figure 9A). Error bars show SD of two biological replicates. **C)** Immunoblotting analyses of H4-K12Ac, H4-K16Ac and H3 (loading control). **D)** DNA 4',6'-diamidino-2-phenylindole (DAPI) staining of representative nuclei and corresponding DAPI coefficient of variation (CV) values. **E)** Boxplots of DAPI CV (n > 110, from one experiment). *DNA compaction statistically different from solvent DMSO control (*P* < 10^-5^, Welch *t*-test). **F)** DNA DAPI staining of representative nuclei from senescent cells that had been treated with TSA for 24 hours and then washed to remove TSA and further incubated with fresh medium for 1 or 6 hours. DMSO: WI-38hTERT/GFP-RAF-ER + 20 nM 4-HT (3 days) + 0.1% dimethyl sulfoxide (DMSO) 24 hours; TSA: WI-38hTERT/GFP-RAF-ER + 20 nM 4-HT (3 days) + 410 nM TSA 24 hours.

The HDAC1 and HDAC2 class I histone deacetylases have also been implicated in regulating global levels of H4-K16Ac [18]. Trichostatin A (TSA) is a potent inhibitor of class I and class II HDACs, but not class III (sirtuins) [39]. Treatment of human fibroblasts with TSA can induce senescence by derepressing CKIs [40]. We thus chose to test the effect of TSA on DNA compaction and H4-K16Ac deacetylation by first inducing senescence by activated RAF1 kinase for 3 days, and then treating the senescent cells with TSA (410 nM) for 24 hours. Histone profiling by mass spectrometry showed that TSA had a remarkably specific effect on augmenting principally mono-acetyl-lysine-H4 under these conditions (Figure 9A,B). Immunoblotting confirmed that this corresponded to an increase in the preponderant acetylation of H4-K16 (Figure 9C). Increased H4-K16Ac in the senescent cells was accompanied by decompaction of DNA (Figure 9D,E). Furthermore, washing out the TSA led to a rapid recompaction of the DNA by 1 hour (Figure 9F). These observations indicate that class I/II HDACs contribute to the dynamic maintenance of H4-K16Ac deacetylation and DNA compaction in senescent cells.

### Oncogene-induced senescence is not universally associated with loss of H4-K16Ac and assembly of striking SAHFs

**Figure 10:**
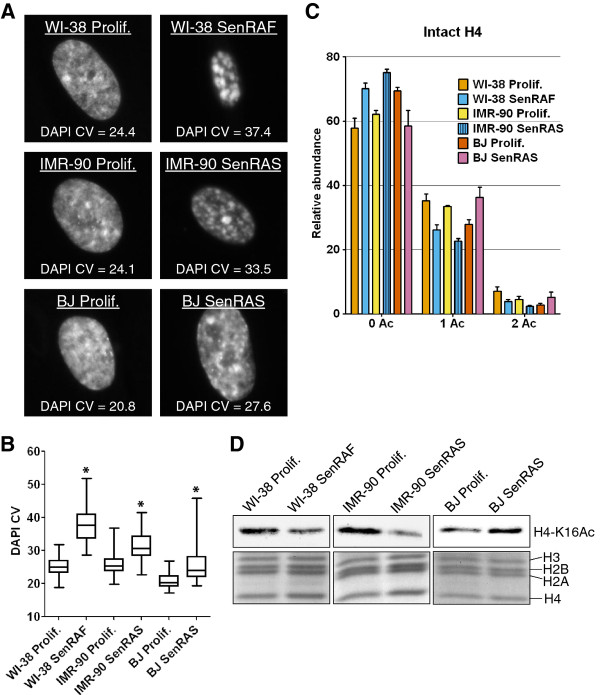
**Oncogene-induced senescence is not universally associated with H4-K16Ac deacetylation and striking senescence-associated heterochromatin foci (SAHFs). A)** DNA 4',6'-diamidino-2-phenylindole (DAPI) staining of representative nuclei and corresponding DAPI coefficient of variation (CV) values. **B)** Boxplots of DAPI CV (n > 60, from one experiment). *DNA compaction statistically different from Prolif. (*P* < 10^-5^, Welch *t*-test). **C)** Relative abundance of H4 acetylation states measured at the protein level on deconvoluted mass spectra. Error bars show SD of at least two biological replicates. **D)** Immunoblot showing the level of H4-K16Ac in acid-extracted histones. Loading control: Coomassie blue staining of histones. WI-38 SenRAF: WI-38hTERT/GFP-RAF-ER + 4-HT 20 nM (5 days); IMR-90 SenRAS: IMR-90/ER-RASval12 + 4-HT 100 nM (6 days); BJ SenRAS: BJ/ER-RASval12 + 4-HT 100 nM (5 days).

It was recently reported that the level of H4-K16Ac could distinguish replicative senescence from RASval12-induced senescence of human BJ foreskin fibroblasts [18]. By immunoblot analyses, replicatively senescent BJ fibroblasts were found to have lower levels of H4-K16 acetylation than proliferating BJ cells, as we observed for WI-38 fibroblasts. However, in contrast to RAF-induced senescence of WI-38, it was reported that RASval12-induced senescence of BJ fibroblasts was associated with an increase rather than a decrease of H4-K16 acetylation compared to proliferating cells. We considered that this difference might be due to the cell line, the hTERT immortalization, or the oncogene used to trigger senescence. We thus measured H4-K16 acetylation in non-immortalized BJ and IMR-90 fibroblasts expressing an ER-RASval12 fusion protein that can trigger senescence after activation of RASval12 by the addition of 4-HT [41]. IMR-90/ER-RASval12 cells senesced with well-developed SAHFs (Figure 10A,B) accompanied by a decrease in H4-K16 acetylation as for RAF-senescent WI-38hTERT cells (Figure 10C,D). In striking contrast, the BJ cells induced into senescence by RASval12 showed increased H4-K16Ac compared to proliferating BJ cells (Figure 10C,D), and the chromatin of RAS-senescent BJ cells was not as compact (Figure 10A,B) as that of RAS-senescent IMR-90 or RAF-senescent WI-38hTERT cells. Narita and colleagues also reported reduced DNA compaction for BJ versus IMR-90 cells induced into senescence by RASval12 [23]. These results indicate that loss of H4-K16Ac is not universally associated with cellular senescence, and the increased H4-K16Ac of BJ cells during RAS-induced senescence is not characteristic of RAS-induced senescence but rather appears to be specific to this cell line. Senescent BJ fibroblasts also express the HMGA2 chromatin protein [24] and the p16 CDK inhibitor [42,43] at lower levels than other fibroblasts. Thus, male BJ foreskin fibroblasts differ by several criteria from female WI-38 or IMR-90 embryonic lung fibroblasts.

**Figure 11:**
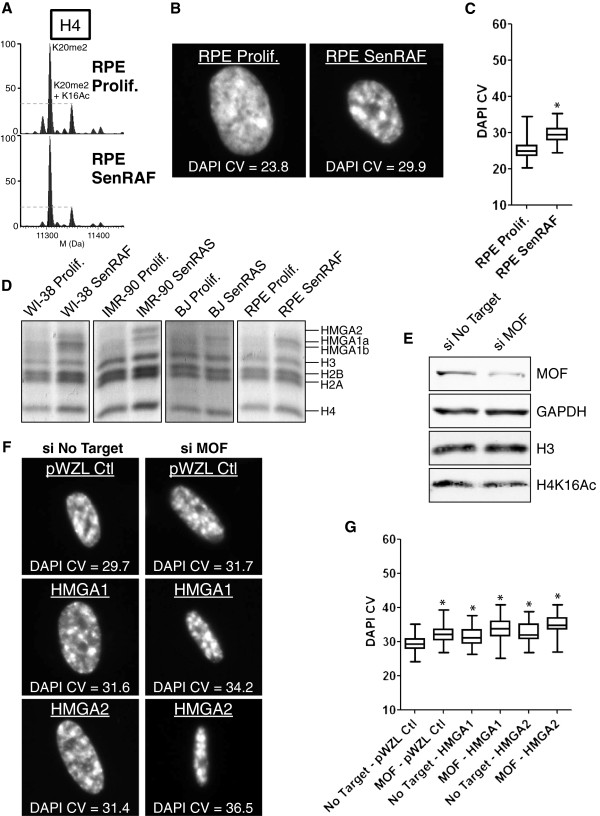
**H4-K16Ac and HMGA proteins act synergistically in DNA compaction leading to the formation of striking senescence-associated heterochromatin foci (SAHFs). A)** Deconvoluted mass spectra of intact histone H4 in proliferating and RAF-senescent RPEhTERT. **B)** DNA 4',6'-diamidino-2-phenylindole (DAPI) staining of representative nuclei and corresponding DAPI coefficient of variation (CV) values. **C)** Boxplots of DAPI CV (n > 80, from one experiment). *DNA compaction statistically different from retinal pigmented epithelial (RPE) Prolif. (*P* < 10^-5^, Welch *t*-test). **D)** SDS-PAGE of acid-extracted proteins from proliferating and oncogene-induced senescent WI-38hTERT, IMR-90, BJ and RPEhTERT cells. **E)** Immunoblot of extracts from RPEhTERT/RAF-ER cells treated with siRNA (24 hours) and 20 nM 4-HT (6 days) using anti-MOF, -GAPDH (loading control), -H4-K16Ac, -H3 (loading control) antibodies. **F)** DNA DAPI staining of representative nuclei and corresponding DAPI CV values. **G)** Boxplots of DAPI CV (n > 50, from one experiment). *DNA compaction statistically different from No Target - pWZL Ctl (*P* < 10^-5^, Welch *t*-test). WI-38 SenRAF: WI-38hTERT/GFP-RAF-ER + 4-HT 20 nM (5 days); IMR-90 SenRAS: IMR-90/ER-RASval12 + 4-HT 100 nM (6 days); BJ SenRAS: BJ/ER-RASval12 + 4-HT 100 nM (5 days); RPE SenRAF: RPEhTERT/GFP-RAF-ER + 4-HT 100 nM (6 days).

Finally, we examined H4-K16Ac levels and DNA compaction during RAF-induced senescence of an hTERT-immortalized retinal pigmented epithelial (RPE) cell line. As for the RAF-induced senescence of WI-38hTERT cells, we observed a decrease in levels of H4-K16Ac during RAF-induced senescence of RPEhTERT cells (Figure 11A). However, DNA compaction was less evident in these cells compared to WI-38hTERT (Figures 11B,C and 10A,B). Chromatin preparations showed that similar to BJ fibroblasts, RAF-senescent RPEhTERT cells contained lower levels of HMGA2 than RAF-senescent WI-38hTERT or RAS-senescent IMR-90 cells (Figure 11D). We explored the effects of H4-K16Ac and HMGA1/2 levels on DNA compaction during RAF-induced senescence of RPEhTERT cells by combining siRNA depletion of MOF with ectopic expression of HMGA1 or HMGA2. Knockdown of MOF decreased the level of H4-K16Ac and increased DNA compaction (Figure 11E,F,G). Ectopic expression of either HMGA1 or HMGA2 also increased DNA compaction. Remarkably, ectopic expression of HMGA1 or HMGA2 in combination with MOF depletion synergistically increased DNA compaction leading to the formation of striking SAHFs (Figure 11F,G). We conclude that deacetylation of H4-K16Ac and expression of HMGA1/2 can both contribute to DNA compaction during senescence.

## Discussion

Deacetylation of H4-K16Ac is associated with chromatin compaction *in vitro*, transcriptional silencing in yeast, and X chromosome inactivation in female humans. We show here that it also occurs during replicative senescence, and is required for the extreme DNA compaction associated with the oncogene-induced senescence (OIS) of human fibroblasts that are competent to assemble SAHFs. In the latter process, SIRT2, but not SIRT1, participates in the global deacetylation of H4-K16Ac.

We showed that oncogene-induced senescence of IMR-90 and WI-38hTERT female human embryonic lung fibroblasts was accompanied by a decrease in H4-K16Ac and highly developed SAHFs. Blocking deacetylation of H4-K16Ac inhibited DNA compaction, but did not noticeably affect the entry or the maintenance of senescence. Stable local repression of cell proliferation genes by the Rb family is clearly required for the entry and maintenance of senescence [23], but this process does not appear to require extensive global compaction of the genome in the form of highly developed SAHFs [42,44]. Surprisingly, the OIS of male BJ foreskin fibroblasts was accompanied by an increase in H4-K16Ac and less highly compacted DNA. We found that levels of global H4-K16Ac depend on a dynamic balance between MOF HAT activity and the deacetylase activities of SIRT2 and TSA-sensitive class I/II enzymes. The TSA sensitive enzymes are probably HDAC1 and HDAC2, which have been previously implicated in contributing to global levels of H4-K16Ac [18]. This dynamic balance thus appears to be regulated in a cell-type specific fashion during OIS even between seemingly very similar fibroblasts. In contrast, replicative senescence of both WI-38 and BJ fibroblasts was accompanied by a decrease in H4-K16Ac. Although morphologically very similar, fibroblasts derived from different anatomical locations retain characteristic epigenetic patterns of gene expression during *in vitro* culture [45]. Differences due to tissue origin or stage of development, gender, or inadvertent *in vitro* selection during the propagation of cell lines may explain the different epigenetic responses of fibroblasts during OIS.

We also found that RAF-induced senescence of retinal pigmented epithelial cells was associated with a decrease in H4-K16Ac, but only low levels of DNA compaction. We found that HMGA2 was poorly expressed in these cells, as is the case for BJ fibroblasts. Remarkably, ectopic expression of HMGA1 or HMGA2 in combination with siRNA depletion of MOF to further decrease H4-K16Ac levels led to a synergistic increase in DNA compaction and the formation of striking SAHFs. Thus, we identified two crucial factors contributing to cell-type specificity in heterochromatin assembly during senescence.

Deregulation of chromatin structure, and H4-K16 acetylation in particular, has been implicated in both aging and cancer. In budding yeast, aging has been associated with a loss of the Sir2 deacetylase and subsequent increase of H4-K16Ac and loss of transcriptional silencing [46]. Overexpression of Sir2 increases the replicative life span of yeast, and this observation led to an explosion of interest in the sirtuin family of NAD-dependent deacetylases. However, the premature aging observed in a progeroid mouse model was associated with hypoacetylation of H4-K16 [47]. Treatment of these mice with the histone deacetylase inhibitor sodium butyrate extended their lifespan. Furthermore, a decrease in mono-acetylated H4 was observed in rat brain cortical neurons during development and aging [48]. Since K16 is the principal acetylated lysine residue of H4 in all studied organisms [49], this observation suggests that loss of H4-K16Ac occurs progressively in aging post-mitotic cortical neurons. We observed a decrease of H4-K16Ac during the replicative senescence of WI-38 fibroblasts that is consistent with a reported decrease during the replicative senescence of BJ fibroblasts [18]. Thus, the aging of human cells subject to telomere attrition may also be associated with loss of this euchromatic mark. These results suggest that heterochromatin formation may increase during aging of mammalian cells, consistent with some studies involving other heterochromatic features, such as H4-K20Me3 [50], and macro-H2A and HP1-beta [51]. However, another study reported a reduction of yet other heterochromatic features (H3-K9Me3 and HP1-gamma) in aging human fibroblasts [52]. These results are not necessarily mutually exclusive and it is possible that aging chromatin is not subject to overall gain or loss of heterochromatin, but rather a more complex set of perturbations that must be characterized at the level of individual marks.

The replicative senescence of budding yeast appears to involve the loss of Sir2 that leads to increases in H4-K16Ac and heterochromatin defects [53]. Depletion of SIRT1, the human sirtuin that is most closely related to Sir2, also accelerates the replicative senescence of human fibroblasts, but this appears to be due to hyperacetylation and activation of the DNA damage checkpoint effector p53 [54]. During RAF-induced senescence of WI-38hTERT fibroblasts, we observed loss of H4-K16Ac that was independent of SIRT1. In contrast, knockdown of SIRT2 prevented loss of H4-K16Ac during RAF-induced senescence and inhibited DNA compaction. SIRT2 is largely cytosolic in interphase cells, but shuttles between the nucleus and cytoplasm [19]. We did not observe a change in its intracellular localization during RAF-induced senescence (unpublished data). SIRT2 has important mitotic functions in deacetylating global H4-K16Ac [16] and in activating the APC (anaphase promoting complex) [55]. SIRT2 activity is inhibited by Cdk2-cyclin A/E phosphorylation [56,57], so that SIRT2 activity may be highest in the M and early G1 phases of cycling cells that do not express these cyclins. During RAF-induced senescence, we observed a slight and early increase in SIRT2 levels that was correlated with an early and progressive loss of H4-K16Ac. It is also likely that SIRT2 activity is increased during senescence by the loss of cyclin A/E whose expression is repressed by the retinoblastoma pathway [58].

Cancer is largely a disease of aging involving the accumulation of genetic and epigenetic modifications that favor uncontrolled growth and metastasis of rogue cells. Interestingly, loss of H4-K16Ac has been reported in cancer cells relative to normal cells [59], although some aspects of this work are controversial and merit further study [13,32]. This observation could be interpreted in several ways. Since cancer arises mainly in the elderly, the decreased H4-K16Ac in cancer cells may reflect loss of H4-K16Ac during the aging of progenitor cells. Another possibility is that decreased H4-K16Ac favors carcinogenesis by decreasing the efficiency of DNA repair and thereby increasing genomic instability [12,14,15,47]. Finally, decreased H4-K16Ac may be related to yet another aspect of the past history of the cancer cell. Expression of mitogenic oncogenes in normal human cells can lead to the induction of cellular senescence as a tumor suppressor mechanism that prevents unscheduled cellular proliferation. Such senescence can be induced in at least two ways. Cancer progenitor cells that do not express telomerase will eventually undergo senescence by loss of telomeric sequences, and we have shown that replicatively senescent cells have decreased H4-K16Ac. In other cell types, hyper-mitogenic signaling induced by oncogene expression induces a rapid senescence even in cells that express telomerase. We showed that such senescence in WI-38hTERT or IMR-90 fibroblasts is also accompanied by a decrease in H4-K16Ac. In both instances, cancer progression requires bypass or escape from senescence [21]. Such cells may nevertheless retain decreased levels of H4-K16Ac. Two papers suggest that cancer cells that escape from senescence may be selected to retain enhanced heterochromatin in order to reduce DNA damage signaling and avoid apoptosis [42,44]. Our observation of reduced H4-K16Ac in many senescent cells, and previous work describing reduced H4-K16Ac in cancer cells [59], would be consistent with this hypothesis. However, this idea was based on the postulate that DNA damage in heterochromatin is poorly detected and inefficiently activates DNA damage checkpoint signaling. Recent data indicate that heterochromatic DNA lesions are in fact detected very efficiently and the damaged DNA is very rapidly transported to the euchromatic boundary for repair [60,61]. It is thus not clear that increased heterochromatin content would protect cancer cells from increased DNA damage signaling engendered by replication stress and genomic instability. Further work should clarify the respective roles of evolutionary history and selective pressures in modulating H4-K16Ac levels and heterochromatin formation in cancer.

## Conclusions

Variable DNA compaction observed during senescence is explained in part by cell-type specific regulation of H4 deacetylation and HMGA1/2 expression. SIRT2 and TSA-sensitive HDACs participate in global deacetylation of H4-K16Ac during RAF-induced senescence, but not SIRT1. Deacetylation of H4-K16Ac during senescence may explain reported decreases in this mark during mammalian aging and in cancer cells.

## Methods

### Cell lines and retroviruses

WI-38hTERT human embryonic fibroblasts expressing a conditionally activated form of the RAF1 kinase (GFP-RAF-ER) were obtained and cultured as described [29]. RPEhTERT/GFP-RAF-ER cells were produced in a similar fashion. WI-38 cells were passaged in ambient 20% oxygen and 5% CO2 to obtain a replicatively senescent population at PD 65. IMR-90 and BJ cells expressing ER-RASval12 were grown as described [41]. Retroviral preparations of pWZL, pWZL-HMGA1, and pWZL-HMGA2 were prepared as described [24].

### Preparation of histones and mass spectrometry analyses

Histones were acid-extracted from various fibroblast cell lines in different conditions and analyzed by MS and MS/MS both at the intact protein and the tryptic peptide levels as previously described [28]. Relative quantification of histone modified forms/variants was measured on deconvoluted ultra-high performance liquid chromatography (UHPLC)-MS spectra by dividing the intensity of a given MS peak by the sum of the intensities of the different MS peaks composing the spectrum of a considered histone. For typtic peptide analyses, histones were first propionylated on lysine residues, then digested with trypsin, and finally subjected to a second round of propionylation to block the newly formed N-terminal residues. Analyses were then performed on a LTQ-Orbitrap Discovery mass spectrometer that was operated in the data-dependent acquisition mode, allowing the automatic switching between MS and MS/MS. The MS survey scan was performed from *m*/*z* 300 *to* 2000 in the Orbitrap, using a resolution set at 30,000 (at *m*/*z* 400). The five most abundant ions (threshold 500 counts, charge states higher than +1) were further selected for collision-induced dissociation (CID) experiments. The CID fragment ions were detected in the linear ion trap. Relative quantification of histone modifications was determined by measuring the area of the extracted ion chromatogram peak corresponding to a specific modified peptide normalized to the sum of the peak areas corresponding to all observed modified forms of this peptide.

### Chemicals

Chemicals were prepared as 1000× stock solutions in the indicated solvents and stored at −20°C. 4-HT (Sigma-Aldrich H6278, Saint Quentin Fallavier, France) was dissolved in ethanol. Etoposide (Sigma-Aldrich E1383), TSA (Sigma-Aldrich T8552), sirtinol (Santa Cruz Biotechnology sc-205976, Santa Cruz, CA), and salermide (Santa Cruz Biotechnology sc-224276 ) were solubilized in DMSO.

### Flow cytometry analyses of DNA content

DNA content analyses were performed with a FACS Calibur flow cytometer [29].

### BrdU incorporation and immunostaining

Cells were seeded in 24-well plates at a density of 50,000 cells/well on collagen-treated coverslips. BrdU was added to media at a final concentration of 50 μM for the indicated periods of time. Immunofluorescence to visualize incorporated BrdU and/or intracellular proteins was performed as described [29] using mouse anti-BrdU (BD Biosciences 555627, San Jose, CA) and rabbit anti-phospho-T68-Chk2 (Cell Signaling Technology 2661, Danvers, MA) under conditions suggested by the manufacturer. We used mouse anti-γH2AX antibodies (Millipore 05–636, Billerica, MA) diluted 1/500 with an incubation of 1 hour at room temperature to visualize γH2AX foci within cells. For DAPI staining, permeabilized cells were treated for 1 minute with 0.5 μg/ml DAPI and then washed with PBS before mounting with Prolong Gold anti-fade medium (Invitrogen). Images of DAPI-stained nuclei were taken with a LEICA DMIRE2 microscope equipped with a 63x/1.4 oil immersion objective, a Roper Instruments CCD camera and Metamorph software (Universal Imaging Corporation Ltd.).

### Quantification of DNA compaction

We designed a plugin for ImageJ that semi-automatically identifies DAPI-stained nuclei and then calculates the CV for DAPI fluorescent intensities for all pixels within each nucleus as a quantitative metric of DNA compaction. We verified that the DAPI CV was independent of exposure time for images that did not contain saturated pixels and that had an empirically determined range of signal intensities. As shown in Figure 1C, we observed a significant difference in CV distribution in proliferating versus RAF-senescent WI-38 fibroblasts. The plugin outputs individual images of all segmented nuclei into an array containing the DAPI CV for all nuclei. The plugin is available upon request.

### Data analysis and statistics

The DAPI CV was calculated for the indicated number of nuclei from one experiment. DAPI CV results are presented as boxplots (with 95% confidence levels and analyzed by the Welch *t*-test). A box represents 50% of the data and the median. Whiskers correspond to the minimum and maximum values. Histone PTM relative abundances were presented on histograms as the average values and SD for the indicated number of biological replicates.

### siRNA transfection

Fibroblasts were transfected with Dharmafect 4 reagent (ThermoFisher Scientific, Surrey, UK) according to the manufacturer’s protocol. We used 100 nM of siGenome SMARTPOOL RNAs for SIRT1 and MOF. SIRT2 was depleted using 50 nM siON-TARGET plus SMARTPOOL and 50 nM siGenome SMARTPOOL RNAs. The control consisted of 100 nM of non-targeting siGenome SMARTPOOL RNAs. Twenty-four hours after transfection, senescence was induced by adding 20 nM 4-HT for 3 days. siRNA knockdown efficiency was confirmed by immunoblotting. To test the effect of SIRT2 mRNA depletion on the maintenance of senescence after CKI knockdown, cells were transfected and induced into senescence as described above. Senescent cells were then transfected with either 100 nM siGenome SMARTPOOL RNAs for p21, 100 nM siRNA sense-strand sequence CCAACGCACCGAAUAGUUA for p16 or both and further cultured in fresh medium without 4-HT for 4 days (BrdU incorporation for 24 hours).

### SDS-PAGE and western blotting

Whole cell extracts were prepared by resuspension of cells in PBS supplemented with anti-protease cocktail (Complete EDTA-free Protease Inhibitor Cocktail Tablets, Roche, Meylan, France), phosphatase inhibitors (10 mM orthovanadate and 20 mM β-glycero-phosphate) and Laemmli 4× sample buffer. Samples were then heat-treated for 10 minutes at 95°C and subsequently sonicated. Protein concentrations were determined by Bradford assay. 10 to 20 μg whole cell protein extracts were separated by 12% or 8% SDS-PAGE and 1 to 2 μg acid-extracted histones were separated by 15% SDS-PAGE. Blots were probed with the following antibodies under manufacturers’ recommendations: rabbit anti-SIRT1 (Santa Cruz Biotechnology sc-15404), mouse anti-SIRT2 (a generous gift from Danny Reinberg’s lab), rabbit anti-MOF (Santa Cruz Biotechnology sc-81765), rabbit anti-GAPDH (Abcam ab9485, Cambridge, United Kingdom), rabbit anti-H3 (Abcam ab1791), rabbit anti-H4-K16Ac (Millipore 07–329), rabbit anti-H4-K12Ac (Millipore 19982), rabbit anti-H4-K8Ac (Millipore 22720). Washed membranes were then probed with secondary antibodies conjugated to infrared dyes, scanned and analyzed with the Odyssey imaging system and its associated software (Li-Cor).

### Quantitative real-time (qRT)-PCR

The efficiency of siRNA depletion of SIRT2 mRNA was assessed by qRT-PCR. 500,000 cells were transfected and senescence was induced 24 hours after transfection for 6 hours by adding 20 nM 4-HT to the cell medium. Total RNA was isolated using a Nucleospin RNA XS kit (Macherey-Nagel, Hœrdt, France). qRT-PCR was performed on a Bio-Rad iQ5 instrument. The reactions were prepared using Platinum SYBR Green qPCR SuperMix-UDG (Invitrogen 11733–046, Cergy Pontoise, France). GAPDH was used as a control gene for normalization. Primer sequences for qRT-PCR: GAPDH-for, ATGGGGAAGGTGAAGGTCG ; GAPDH-rev, GGGGTCATTGATGGCAACAATA ; SIRT2-for, AGGCCAAGGCTTAAACAGGCATC ; SIRT2-rev, TCCTTAGCCCAGGAGTGG TTAGAG.

## Abbreviations

APC: anaphase promoting complex; BrdU: 5-bromo-2'-deoxyuridine; CID: collision-induced dissociation; CKI: cyclin-dependent kinase inhibitor; CV: coefficient of variation; DAPI: 4',6'-diamidino-2-phenylindole; DMSO: dimethyl sulfoxide; GFP: green fluorescent protein; HAT: histone acetyl transferase; HDAC: histone deacetylase; 4-HT: 4-hydroxy-tamoxifen; MS: mass spectrometry; OIS: oncogene-induced senescence; PBS: phosphate buffered saline; PTMs: post-translational modifications; PD: population doubling; RPE: retinal pigmented epithelial; SA-ß-gal: senescence-associated ß-galactosidase; SAHF: senescence-associated heterochromatin foci; SD: standard deviation; SIR: silent information regulator; TSA: trichostatin A; UHPLC: ultra-high performance liquid chromatography.

## Competing interests

The authors declare that they have no competing interests.

## Authors' contributions

KC performed most of the experiments with assistance from RC in experiments involving lentiviruses. JYT developed the ImageJ plugin to quantify DNA compaction and performed statistical analyses. FF supervised all the MS analyses, and CM and KC designed the study and wrote most of the manuscript. All authors contributed to and approved the final version of the manuscript.
